# Practice Effect of Repeated Cognitive Tests Among Older Adults: Associations With Brain Amyloid Pathology and Other Influencing Factors

**DOI:** 10.3389/fnagi.2022.909614

**Published:** 2022-07-06

**Authors:** Bang Zheng, Chinedu Udeh-Momoh, Tamlyn Watermeyer, Celeste A. de Jager Loots, Jamie K. Ford, Catherine E. Robb, Parthenia Giannakopoulou, Sara Ahmadi-Abhari, Susan Baker, Gerald P. Novak, Geraint Price, Lefkos T. Middleton

**Affiliations:** ^1^Ageing Epidemiology (AGE) Research Unit, School of Public Health, Imperial College London, London, United Kingdom; ^2^Department of Non-communicable Disease Epidemiology, London School of Hygiene & Tropical Medicine, London, United Kingdom; ^3^Edinburgh Dementia Prevention, Centre for Clinical Brain Sciences, University of Edinburgh, Edinburgh, United Kingdom; ^4^Janssen Research and Development LLC, Titusville, NJ, United States; ^5^Public Health Directorate, Imperial College NHS Healthcare Trust, London, United Kingdom

**Keywords:** practice effect, cognitive test, older adults, amyloid pathology, memory

## Abstract

**Background:**

Practice effects (PE), after repeated cognitive measurements, may mask cognitive decline and represent a challenge in clinical and research settings. However, an attenuated practice effect may indicate the presence of brain pathologies. This study aimed to evaluate practice effects on the Repeatable Battery for the Assessment of Neuropsychological Status (RBANS) scale, and their associations with brain amyloid status and other factors in a cohort of cognitively unimpaired older adults enrolled in the CHARIOT-PRO SubStudy.

**Materials and Methods:**

502 cognitively unimpaired participants aged 60-85 years were assessed with RBANS in both screening and baseline clinic visits using alternate versions (median time gap of 3.5 months). We tested PE based on differences between test and retest scores in total scale and domain-specific indices. Multiple linear regressions were used to examine factors influencing PE, after adjusting for age, sex, education level, *APOE*-ε4 carriage and initial RBANS score. The latter and PE were also evaluated as predictors for amyloid positivity status based on defined thresholds, using logistic regression.

**Results:**

Participants’ total scale, immediate memory and delayed memory indices were significantly higher in the second test than in the initial test (Cohen’s d_z_ = 0.48, 0.70 and 0.35, *P* < 0.001). On the immediate memory index, the PE was significantly lower in the amyloid positive group than the amyloid negative group (*P* = 0.022). Older participants (≥70 years), women, non-*APOE*-ε4 carriers, and those with worse initial RBANS test performance had larger PE. No associations were found between brain MRI parameters and PE. In addition, attenuated practice effects in immediate or delayed memory index were independent predictors for amyloid positivity (*P* < 0.05).

**Conclusion:**

Significant practice effects on RBANS total scale and memory indices were identified in cognitively unimpaired older adults. The association with amyloid status suggests that practice effects are not simply a source of measurement error but may be informative with regard to underlying neuropathology.

## Introduction

Valid instruments and implementations of cognitive tests are essential for the evaluation of cognitive status, decline and subsequent dementia diagnosis, and the screening of at-risk participants for clinical trials and population intervention programs for dementia prevention. However, practice effects (PE) after repeated cognitive measurements, which refer to improvements in test performance due to repeated exposure to test materials or procedures (Hausknecht et al., 2007; Goldberg et al., 2015), often mask a potential cognitive decline and remain a major issue in clinical and research settings (Houx et al., 2002; Sanderson-Cimino et al., 2022). Failing to account for practice effects in cognitive tests could delay diagnosis and clinical care for patients with cognitive deficits. PE resulting from task familiarity occurring with test repetition is distinct from learning effects which refer to the recall of correct answers from previous tests. The latter is often addressed in neuropsychological practice through administration of alternate versions of the same task (e.g., different word lists in verbal memory tests).

Exploring factors that influence practice effects can be informative of potential heterogeneity of measurement bias and in developing mitigation strategies to minimise such bias (Calamia et al., 2012). On the other hand, the magnitude of practice effect *per se* may also have indicative value for cognitive impairment or existing brain pathologies (Duff et al., 2007; Jutten et al., 2021). From this perspective, PE may represent not merely a source of measurement error but potentially valuable information from a clinical and scientific perspective (Duff et al., 2007).

Given the long preclinical stage of late-onset dementia (Elias et al., 2000) with progressively accumulating neuropathology, it is early detection in at-risk individuals that may prove essential in reducing the burden of cognitive and functional decline and dementia in the elderly population. Therefore, a deeper understanding and characterisation of PE in validated cognitive assessment tools among asymptomatic population is warranted.

This study aimed to evaluate PE in the Repeatable Battery for the Assessment of Neuropsychological Status (RBANS) (Randolph et al., 1998), and its associations with brain amyloid status and other factors in a cohort of cognitively unimpaired older adults in the United Kingdom Cognitive Health in Ageing Register: Investigational, Observational, and Trial Studies in Dementia Research: Prospective Readiness cOhort Study (CHARIOT-PRO) SubStudy (Udeh-Momoh et al., 2021).

## Materials and Methods

### Study Population

CHARIOT-PRO SubStudy is an on-going prospective cohort study of cognitively unimpaired older adults in the United Kingdom, which aims to examine longitudinal cognitive changes in those with and without brain amyloid-beta (Aβ42) pathology, and factors and markers of subsequent decline (Udeh-Momoh et al., 2021). Following screening of 2425 individuals, including amyloid status determination and multiple cognitive tests, an equal number of participants above and below a binary threshold of Aβ42 positivity were enrolled at baseline and in subsequent longitudinal study. During screening, participants whose performance on any RBANS index was poorer than 1.5 standard deviation (SD) below the population mean (population norms from Randolph, 1998) were referred to an adjudication panel of neurologists, psychiatrists and neuropsychologists to detect any undiagnosed cognitive impairment which was an exclusion criterion. The detailed inclusion/exclusion criteria and study procedures have been described in previous papers of our group (Nalder et al., 2021; Udeh-Momoh et al., 2021). The study received approval from the National Research Ethics Service (NRES) Committee London Central [reference 15/LO/0711 (IRAS 140764)], as well as independent ethics review by committees from the local sites. All participants provided informed consent before participating in the study.

A total of 502 participants aged 60–85 years completed RBANS assessments in both screening and baseline clinic visits and were included in this study (Udeh-Momoh et al., 2021). The median time gap between the screening visit and the baseline visit was 3.5 months, which allowed us to examine the practice effects in RBANS scale within a relatively short time period with less concern that the test-retest score differences are (partially) due to the cognitive decline during this time interval.

### Measurements

Repeatable Battery for the Assessment of Neuropsychological Status (RBANS) (Randolph et al., 1998) is a validated and widely used neuropsychological assessment. It is a 20-min composite battery which consists of twelve subtests that measure five cognitive domain indices (immediate memory, delayed memory, visuospatial construction, language, attention). The sum of the five index scores is converted to a total scale score based on a distribution with a mean of 100 and SD of 15. This assessment was administered by trained assistant psychologists during the in-person clinic visits. Version C and Version A of the RBANS were administered at the screening and baseline assessments, respectively, to avoid learning effects (i.e., recalling answers from the same test received before).

Amyloid burden was determined during the screening visit either by amyloid positron emission tomography (PET) scans (in ∼90% of participants) or cerebrospinal fluid (CSF) Aβ42 measurements via lumbar punctures (in the remaining 10%). Aβ positive was defined as above-threshold brain Aβ deposition on PET (based on tracer-specific thresholds of the composite cortical standardised uptake value ratio, SUVR) or below-threshold CSF Aβ42 concentration (≤600 ng/L). Three F18-radiolabeled amyloid tracers were used: florbetapir (Amyvid), flutemetamol (Vizamyl) and florbetaben (Neuraceq). The composite cortical SUVR threshold was 1.14 for Amyvid and 1.23 for Vizamyl (both with whole cerebellum as reference region), and 1.20 for Neuraceq (with cerebellar grey matter as reference region) (Udeh-Momoh et al., 2021).

Screening also included a brain magnetic resonance imaging (MRI). Bilateral volumetric MRI parameters were obtained, including whole brain volume (mL^3^), ventricular volume (mL^3^), hippocampal volume (mm^3^) and AD signature cortical thickness (mm) (Schwarz et al., 2016). Intracranial volume (ICV) was used as the proxy variable for premorbid brain volume to be adjusted for in the analyses of MRI parameters. All study procedures and cut-off points have previously been reported (Udeh-Momoh et al., 2021).

We also collected other information including age, sex, ethnicity, education level, *APOE* genotype and National Adult Reading Test (NART) score [as a proxy for premorbid intelligence quotient (IQ)] (Nelson and Willison, 1991).

### Statistical Analyses

Demographic and clinical characteristics of study participants were compared according to amyloid pathology status (amyloid positive vs. negative) using independent samples *t*-test, chi-squared test, rank-sum test or general linear regression, where appropriate. We assessed the internal consistency reliability (Cronbach’s α coefficient) and test-retest reliability (Pearson correlation coefficient *r*) of the RBANS scale in this cohort. PE was estimated based on differences between test and retest scores (i.e., measurements at the screening and baseline visits) in RBANS total scale and domain-specific indices. Paired *t*-test was used to test the statistical significance of PE; Cohen’s d_z_ for the within-subjects design (Cohen, 1988) was calculated as the standardised effect size for PE (i.e., scaled difference scores).

Multiple linear regression model was used to examine whether the magnitude of PE varies by amyloid status, with the test-retest difference score in RBANS total scale or domain-specific index as the dependent variable, amyloid status as the independent variable of interest, while adjusting for age, sex, education level, *APOE*-ε4 carriage and initial RBANS level. Following the same procedure, we also explored other potential influencing factors of PE in separate linear regression models, including age group (60–69 years vs. 70–85 years), sex, education level (below/above upper secondary education), *APOE*-ε4 (carrier vs. non-carrier), test-retest time interval (1-3 months vs. 4-6 months), MRI parameters (below/above mean), National Adult Reading Test score (below/above median), and initial RBANS scores (below/above mean).

To assess the robustness of our main findings, we conducted the following sensitivity analyses: (1) modelling MRI parameters, age, test-retest time interval, initial RBANS score and NART score as continuous variables instead of dichotomised variables when exploring their associations with PE; (2) excluding 52 participants who waited for over 6 months after the screening visit to attend the baseline visit to avoid the loss of PE or occurrence of possible cognitive decline during the prolonged time gap; (3) additionally adjusting for test-retest time interval and modality of amyloid (PET or CSF) when assessing the amyloid-PE association.

Finally, to explore the predictive value of PE, PE was also assessed as a predictor together with initial RBANS score for amyloid positive status using binary logistic regression, adjusting for age, sex, education level, and *APOE*-ε4 carriage. The odds ratio (OR) and 95% confidence interval (CI) of standardised PE scores (i.e., centred and scaled) was reported, which reflects the relative risk of the presence of amyloid pathology per 1 SD increase in PE.

Statistical analyses were conducted using Stata (version 15; College Station, TX: StataCorp LLC). All statistical analyses are two-sided. A *P* value of < 0.05 indicates a statistically significant result.

## Results

### Population Characteristics

Of the 502 participants assessed with RBANS scale in both screening and baseline clinic visits with median time gap of 3.5 months (interquartile range: 2.9–4.4), the mean (SD) age was 71.4 (5.5) years, and 254 (50.6%) were females. 192 participants (38.2%) were *APOE*-ε4 carriers and 247 (49.2%) were Aβ positive based on CSF Aβ42 level or PET scans. Nearly all participants (95.8%) were White. Most participants (85.7%) had completed upper secondary education or above.

Participant characteristics are presented by amyloid pathology status in Table 1. Aβ+ participants were slightly older and more likely to be *APOE*-ε4 carriers compared with Aβ- participants (*P* < 0.05). Differences in MRI parameters were also observed between amyloid groups, with Aβ+ group having lower hippocampal volume, whole brain volume, and AD signature cortical thickness (*P* < 0.05). The RBANS test-retest time interval was similar between Aβ+ group and Aβ- group (*P* = 0.728).

**TABLE 1:** Population characteristics by amyloid status (*N* = 502).

Characteristics	Total	Amyloid positive	Amyloid negative	*P*-value
N	502	247	255	
Age (years), $\bar{x}$ ± SD	71.4 ± 5.5	72.3 ± 5.6	70.4 ± 5.4	< 0.001
Female, %	50.6	48.6	52.6	0.374
Ethnicity (White), %	95.8	96.8	94.9	0.298
Below upper secondary education, %	14.3	17.0	11.8	0.094
*APOE*-ε4 carrier, %	38.2	54.7	22.4	< 0.001
NART score, $\bar{x}$ ± SD	9.9 ± 6.7	9.5 ± 5.9	10.3 ± 7.3	0.202
Days between test and retest, median (IQR)	107 (87–133)	106 (86–133)	108 (87–136)	0.728
RBANS score (first test), $\bar{x}$ ± SD				
Total scale	102.7 ± 11.8	102.6 ± 11.7	102.9 ± 11.9	0.734
Immediate memory index	101.6 ± 12.7	101.0 ± 12.3	102.2 ± 13.0	0.268
Delayed memory index	100.7 ± 10.1	99.8 ± 10.9	101.6 ± 9.2	0.045
Visuospatial construction index	95.7 ± 14.1	96.7 ± 13.9	94.9 ± 14.3	0.148
Language index	104.1 ± 11.5	104.5 ± 11.0	103.7 ± 12.0	0.422
Attention index	108.8 ± 14.5	108.5 ± 13.8	109.2 ± 15.2	0.607
MRI parameters, $\bar{x}$ ± SD				
Hippocampal volume (mm^3^)	7754 ± 852	7621 ± 899	7883 ± 794	< 0.001
Whole brain volume (mL^3^)	1094629 ± 107552	1087603 ± 109462	1101408 ± 105861	0.005
Ventricular volume (mL^3^)	35701 ± 16987	36381 ± 16991	35045 ± 16886	0.304
AD signature cortical thickness (mm)	2.80 ± 0.12	2.79 ± 0.13	2.81 ± 0.12	0.028

*SD, standard deviation; NART, National Adult Reading Test; IQR, interquartile range; RBANS, Repeatable Battery for the Assessment of Neuropsychological Status; MRI, magnetic resonance imaging; AD, Alzheimer’s disease. P-values were calculated by chi-squared tests, t-tests, rank-sum test, or general linear regressions to adjust for intracranial volume for volumetric MRI parameters.*

### Practice Effects in Repeatable Battery for the Assessment of Neuropsychological Status Assessment

The internal consistency reliability of RBANS scale in our study sample measured by Cronbach’s α was 0.64, and the test-retest reliability measured by Pearson correlation coefficient *r* was 0.79.

Participants had significantly higher scores in RBANS total scale and immediate and delayed memory indices in the second test than in the initial test (increased score = 3.9, 7.6, and 3.3, respectively; *P* < 0.001; Table 2). After taking into account the differences in variances of these indices, the calculation of within-subject Cohen’s d_z_ revealed a strong effect size for PE in immediate memory index (0.70), and a low-to-moderate effect size for PE in RBANS total scale (0.48) and delayed memory index (0.35). In contrast, no significant PEs were identified for the rest of the three domain indices (Cohen’s d_z_ ranged from 0.05 to 0.06; *P* > 0.05; Table 2).

**TABLE 2:** Differences between test and retest performance in repeatable battery for the assessment of neuropsychological status (RBANS) (*N* = 502).

RBANS score, *x̄* ± SD	Test	Retest	Difference score (mean)	Difference score (range)	Cohen’s d_z_	*P*-value
Total scale	102.7 ± 11.8	106.6 ± 12.9	3.9	–20, 38	0.48	< 0.001
Immediate memory index	101.6 ± 12.7	109.2 ± 13.4	7.6	–28, 35	0.70	< 0.001
Delayed memory index	100.7 ± 10.1	104.0 ± 10.6	3.3	–35, 36	0.35	< 0.001
Visuospatial construction index	95.8 ± 14.1	96.6 ± 14.3	0.8	–37, 41	0.06	0.176
Language index	104.1 ± 11.5	104.8 ± 13.0	0.7	–42, 41	0.06	0.209
Attention index	108.8 ± 14.5	109.3 ± 14.7	0.5	–31, 32	0.05	0.293

*RBANS, Repeatable Battery for the Assessment of Neuropsychological Status; SD, standard deviation. P-values were calculated by paired t-tests.*

### Practice Effects in Repeatable Battery for the Assessment of Neuropsychological Status by Amyloid Pathology Status

**FIGURE 1:**
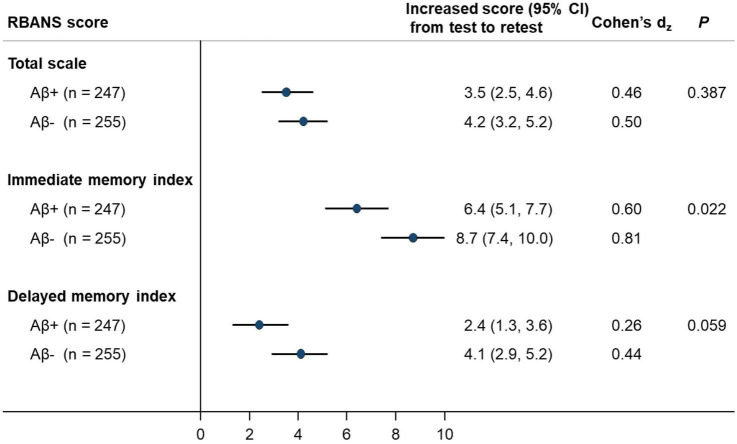
Associations between amyloid status with magnitude of RBANS practice effects (*N* = 502). RBANS, Repeatable Battery for the Assessment of Neuropsychological Status; CI, confidence interval. Estimates were adjusted for age, sex, education level, *APOE*-ε4 carriage and initial RBANS level, where applicable.

We examined the practice effects in RBANS total scale and memory indices by amyloid pathology status (Figure 1). After adjusting for potential confounding factors, the amyloid positive group had significantly lower PE in immediate memory index than the amyloid negative group (Cohen’s d_z_ = 0.60 vs. 0.81; *P* = 0.022). Similarly, a borderline statistical significance was observed for lower PE in delayed memory index, in the amyloid positive group (Cohen’s d_z_ = 0.26 vs. 0.44; *P* = 0.059). However, the difference in PE in RBANS total scale by amyloid status did not reach statistical significance (Cohen’s d_z_ = 0.46 vs. 0.50; *P* = 0.387; Figure 1). We also generated spaghetti plots by amyloid status to visualise the heterogeneity in practice effects across individuals (Supplementary Figures 1–3).

### Other Influencing Factors on Practice Effect in Repeatable Battery for the Assessment of Neuropsychological Status

In the exploratory analyses for brain MRI parameters and PE, we observed no significant associations of hippocampal volume, whole brain volume, ventricular volume or AD signature cortical thickness with the magnitude of PE in RBANS total scale or memory indices (Supplementary Table 1).

Older adults (≥70 years), women, and *APOE*-ε4 non-carriers had larger PE in one or more RBANS indices (*P* < 0.05; Table 3). Those with worse performance in the initial RBANS test had larger PE in both total scale and the individual memory indices (*P* < 0.05; Table 3). Test-retest time interval, education level and NART score had no significant association with the magnitude of PE (Supplementary Table 1). Sensitivity analyses revealed consistent results with the main findings (Supplementary Tables 2–5).

**TABLE 3:** Associations between other characteristics and magnitude of repeatable battery for the assessment of neuropsychological status (RBANS) practice effects (*N* = 502).

Characteristics	No. of participants	Increase of total scale (95% CI)	*P*-value	Increase of immediate memory index (95% CI)	*P*-value	Increase of delayed memory index (95% CI)	*P*-value
Age (years)			0.565		0.146		0.009
60–69	208	4.1 (3.0, 5.2)		6.8 (5.3, 8.2)		2.0 (0.8, 3.2)	
70–85	294	3.7 (2.8, 4.6)		8.1 (6.9, 9.3)		4.2 (3.1, 5.2)	
Sex			0.018		0.002		0.712
Male	248	3.0 (2.0, 4.0)		6.1 (4.8, 7.4)		3.1 (2.0, 4.2)	
Female	254	4.7 (3.7, 5.7)		9.0 (7.7, 10.2)		3.4 (2.3, 4.6)	
*APOE*-ε4			0.164		0.004		0.337
Carrier	192	3.2 (2.1, 4.4)		5.9 (4.4, 7.3)		3.8 (2.5, 5.0)	
Non-carrier	310	4.3 (3.4, 5.2)		8.6 (7.5, 9.8)		3.0 (2.0, 4.0)	
Initial RBANS score			0.002		< 0.001		< 0.001
Higher than mean level	238	2.7 (1.6, 3.7)		4.7 (3.5, 6.0)		0.9 (–0.2, 1.9)	
Lower than mean level	264	5.0 (4.0, 5.9)		10.7 (9.3, 12.0)		6.2 (5.0, 7.4)	

*RBANS, Repeatable Battery for the Assessment of Neuropsychological Status; CI, confidence interval. Estimates were adjusted for age, sex, education level, APOE-ε4 carriage and initial RBANS level, where applicable.*

### Attenuated Practice Effect Is Indicative of Above Threshold Amyloid Pathology

We further explored the indicative value of PE for brain amyloid pathology. Results of multiple logistic regressions showed that, besides age (OR = 1.09, 95% CI: 1.05–1.13 per year) and *APOE*-ε4 carriage (OR = 5.50, 95% CI: 3.60–8.40), worse initial performance and lower PE in delayed memory index were independent predictors for amyloid positivity, with similar magnitudes of association (OR per 1 SD increase = 0.78, 95% CI: 0.63-0.97). As for immediate memory, lower PE (OR = 0.75, 95% CI: 0.61–0.94) but not performance in the initial test (OR = 0.82, 95% CI: 0.66–1.02) was a significant predictor for amyloid positivity. We did not find an association between PE in RBANS total scale and existing amyloid pathology (OR = 0.92, 95% CI: 0.75–1.12).

## Discussion

In this prospective cohort study of cognitively unimpaired older adults, enriched with fluid and neuroimaging biomarker data, we comprehensively assessed the practice effect in RBANS assessment and its potential influencing factors, with a focus on brain amyloid pathology. We observed significant practice effects for RBANS total scale and two memory indices, where participants performed better after repeated measurement using alternate versions of these tasks. The magnitude of practice effects differed by amyloid pathology status, age, sex, *APOE*-ε4 carriage and initial RBANS scores, but had no association with brain MRI parameters, education level or NART score.

Our findings suggest that PE in cognitive tests may be domain-specific. Of the five cognitive domains assessed by RBANS scale, only the two memory indices presented significant practice effects, whilst participants’ performance in visuospatial construction, language and attention domains remained similar between the first and second tests over a median of 3.5 months. Our results were in line with a previous study of a much smaller sample of 36 healthy adults (Bartels et al., 2010), where clinically relevant PE was observed during high-frequency testing within three months in learning and memory tests but not in language and visuospatial tests. Similarly, a study of 947 cognitively normal older adults from the Mayo Clinic Study of Aging showed large PE in learning and memory tests but low PE in language tests, using the Mayo Clinic neurocognitive battery (Machulda et al., 2013).

Regarding the memory domain indices, we observed a much larger effect size of PE for immediate memory index than that for delayed memory index or the RBANS total scale. This implies that PE may be more pronounced in immediate memory tasks where people tend to get better at doing these tasks following familiarisation with the test materials or procedures, even when assessed with different word lists (Houx et al., 2002). Thus, the immediate memory test seems to be a more sensitive measure of PE, compared with other domains or the global composite score. The contrast between immediate and delayed memory PEs might alternatively reflect differences in the content of the measures. Specifically, the RBANS immediate memory index is derived solely from tests of verbal recall, whereas the delayed memory index also incorporates verbal recognition and visual-constructional recall. Future systematic evaluation of practice effects in individual test scores rather than the overall indices, with larger sample size and careful control of multiple testing, may help identify even more sensitive metrics.

Our data are in line with previous reports, suggesting the predictive value of PE for the presence of amyloid pathology and subsequent cognitive decline, in addition to merely evaluating cognitive measurement. To be noted, on average, the RBANS scores in our study participants were within “cognitively healthy” boundaries, even in the amyloid positive group and would not prompt further testing in a clinical scenario. This observation underscores the potential value of diminished practice effects as an adjunct metric to traditional assessments for the sensitive detection of preclinical AD. Several previous studies have consistently shown that diminished PE over repeated cognitive testing (mainly episodic memory measures) was associated with subsequent cognitive decline and increased risk of mild cognitive impairment (MCI) or dementia (Duff et al., 2007; Sanchez-Benavides et al., 2016; Jutten et al., 2020, 2021). In contrast, previous evidence on the association between PE and AD biomarkers and neuropathology remained inconsistent (Duff et al., 2018; Ihara et al., 2018; Jutten et al., 2020). A previous systematic review on PE in cognitive assessment identified four papers reporting an association between higher amyloid uptake on amyloid PET scans and lower PE, whereas two papers did not detect this association (Jutten et al., 2020). In our study, the attenuated PE in memory indices was associated with the presence of high amyloid burden but not with brain MRI features, including hippocampal volume, implying that PE in memory tests could be more indicative of β-amyloidosis [which is specific for Alzheimer’s disease (AD)] instead of biomarkers of neurodegeneration or neuronal injury (Jack et al., 2016). Consistent with our results, a recent report from the Harvard Aging Brain Study, of 114 cognitively unimpaired older adults, showed that lower PE in a self-administered computerised cognitive composite battery over the first 3 months was associated with more global amyloid burden (based on PiB-PET imaging) and tau deposition in the entorhinal cortex and inferior-temporal lobe (based on Flortaucipir PET imaging) (Jutten et al., 2021). These findings imply the usefulness of PE as an early detection tool for signs of disease burden prior to the emergence of cognitive impairment, which might inform participant stratification and biomarker testing strategies for clinical trials.

In our exploratory analyses, practice effects in RBANS total scale or memory indices were more pronounced in older adults, women, *APOE*-ε4 non-carriers and those with worse performance in the initial RBANS assessment (probably due to larger room for improvement). Of note, these factors were associated with different indices, indicating a complex domain-specific PE population heterogeneity. Our finding of a positive association between age and PE was inconsistent with a previous meta-analysis report (Calamia et al., 2012) of a negative association, in a much younger population (mean age of around 40 to 50 years). In the afore-mentioned Mayo Clinic report (Machulda et al., 2013), no significant PE differences were found on memory test scores between those aged below and above 80 years. A previous systematic review identified three papers reporting an association between presence of ≥ 1 *APOE*-ε4 allele and lower PE, whereas three papers did not detect this association (Jutten et al., 2020). Further studies are warranted to elucidate the nature and extent of these population heterogeneities in PE, which could be crucial for clinical trials in obtaining unbiased effect estimate for tested treatment or intervention. If the factors affecting PE are not well balanced between placebo and treatment groups, the two groups may have different levels of PE, in which case researchers need to control for these factors so that the estimate of difference in cognitive outcomes between groups can be attributed to treatment.

The availability of extensive phenotypic (including fluid and neuroimaging biomarker) data is a key strength of our study. Moreover, the relatively short test-retest interval (median of 3.5 months) was essential in minimising the risk of a potential cognitive decline during the test-retest interval affecting the presence and extent of PE. If given a long test-retest period, PE may be masked by progressive cognitive decline over time and it would be difficult to distinguish one from the other.

Several limitations need to be taken into consideration when interpreting our results. Since we explored multiple influencing factors on PE in our study, the risk of inflated Type 1 error in multiple testing cannot be ruled out. Therefore, our exploratory analyses need further validation. Moreover, RBANS does not provide an isolated scale of executive function, a domain which has been independently associated with early amyloidosis rather than memory performance decrements in cognitively normal adults (Tideman et al., 2022). Assessing diminished practice effects in this domain may yet provide even more sensitive markers of subtle cognitive signs. Due to the different modalities and tracers used for amyloid testing in this study, we did not evaluate the amyloid pathology on a quantitative scale which is worth to be considered in future studies. In addition, we only used data from two time points; future studies on longitudinal PE across multiple measurements (with short between-test intervals) are needed. For instance, it is worth exploring whether the PE beyond the second test is not as large as that between the first two tests, which may have important implications for research and clinical purposes (e.g., recommending the second assessment to be considered as baseline measure to minimise PE in outcome assessment). Furthermore, since our test-retest time gap mainly fell between 3 and 4 months, future large-scale studies with time gaps of wider distribution could provide insights for what might be too short vs. too long for detecting PE, though it is possible that the optimal time gap could be different for different cognitive domains or tasks. Finally, our study population are cognitively unimpaired older adults; it would also be interesting to investigate PE in MCI or AD patients, which may show different profiles (Machulda et al., 2013). Similarly, the study sample lacks ethnic and racial diversity (95.8% White people) thereby limiting the generalisability of our findings.

In conclusion, we identified significant PE in RBANS total scale and memory indices among a cohort of cognitively unimpaired older adults. PE is not simply a source of measurement bias in cognitive assessment, but may be informative with regard to a significant brain amyloid pathology burden.

## Data Availability Statement

The datasets presented in this article are not readily available at present due to embargo on the data. Requests to access the datasets should be directed to the corresponding author.

## Ethics Statement

This study received approval from the National Research Ethics Service (NRES) Committee London Central (reference 15/LO/0711 (IRAS 140764)), as well as independent ethics review by committees from the local sites. All participants provided informed consent before participating in the study.

## Author Contributions

GP, LM, CU-M, BZ, and TW contributed to study design and conception. BZ and CU-M carried out data analysis and interpretation. BZ, LM, and CU-M drafted the first version of the manuscript. All authors critically reviewed and revised the manuscript.

## Conflict of Interest

SB and GN were employed by Janssen Research and Development LLC. The remaining authors declare that the research was conducted in the absence of any commercial or financial relationships that could be construed as a potential conflict of interest. The authors declare that this study received funding from Janssen Research & Development, USA. This is a collaborative study with the sponsor’s clinicians and scientists.

## Publisher’s Note

All claims expressed in this article are solely those of the authors and do not necessarily represent those of their affiliated organizations, or those of the publisher, the editors and the reviewers. Any product that may be evaluated in this article, or claim that may be made by its manufacturer, is not guaranteed or endorsed by the publisher.
