# Supplementary material for: Practice Effect of Repeated Cognitive Tests Among Older Adults: Associations With Brain Amyloid Pathology and Other Influencing Factors
Source: Front Aging Neurosci. 2022 Jul 6;14:909614. doi: 10.3389/fnagi.2022.909614 (PMC9297730; doi:10.3389/fnagi.2022.909614)
Supplement: Supplementary file 1 [file Data_Sheet_1.docx]

Supplementary Material

**Supplementary Table 1. Associations of participant characteristics with magnitude of RBANS practice effects**

| **Characteristics** | **No. of participants** | **Increase of total scale (95% CI)** | ***P* value** | **Increase of immediate memory index (95% CI)** | ***P* value** | **Increase of delayed memory index (95% CI)** | ***P* value** |
| --- | --- | --- | --- | --- | --- | --- | --- |
| Education level |  |  | 0.164 |  | 0.116 |  | 0.894 |
| Below upper secondary education | 72 | 2.6 (0.8, 4.5) |  | 5.8 (3.4, 8.2) |  | 3.4 (1.3, 5.5) |  |
| Upper secondary education and above | 430 | 4.1 (3.3, 4.8) |  | 7.9 (6.9, 8.8) |  | 3.2 (2.4, 4.1) |  |
| NART score |  |  | 0.364 |  | 0.198 |  | 0.133 |
| Below median | 250 | 4.2 (3.2, 5.3) |  | 8.2 (6.8, 9.5) |  | 3.9 (2.7, 5.0) |  |
| Above median | 250 | 3.5 (2.4, 4.5) |  | 6.9 (5.6, 8.2) |  | 2.6 (1.4, 3.7) |  |
| Test-retest time interval |  |  | 0.257 |  | 0.161 |  | 0.941 |
| 1-3 months | 137 | 3.4 (2.1, 4.7) |  | 6.6 (4.9, 8.3) |  | 3.4 (1.9, 4.9) |  |
| 4-6 months | 313 | 4.3 (3.5, 5.2) |  | 8.1 (7.0, 9.2) |  | 3.5 (2.5, 4.4) |  |
| Hippocampal volume |  |  | 0.954 |  | 0.146 |  | 0.385 |
| Below mean | 248 | 3.9 (2.8, 4.9) |  | 6.8 (5.4, 8.1) |  | 2.9 (1.7, 4.0) |  |
| Above mean | 254 | 3.8 (2.8, 4.9) |  | 8.3 (6.9, 9.6) |  | 3.6 (2.5, 4.8) |  |
| Whole brain volume |  |  | 0.381 |  | 0.791 |  | 0.356 |
| Below mean | 263 | 3.4 (2.2, 4.6) |  | 7.4 (5.8, 8.9) |  | 3.8 (2.4, 5.1) |  |
| Above mean | 239 | 4.3 (3.1, 5.6) |  | 7.7 (6.1, 9.4) |  | 2.7 (1.2, 4.1) |  |
| Ventricular volume |  |  | 0.286 |  | 0.914 |  | 0.906 |
| Below mean | 292 | 3.5 (2.5, 4.5) |  | 7.5 (6.2, 8.8) |  | 3.2 (2.1, 4.3) |  |
| Above mean | 210 | 4.4 (3.2, 5.6) |  | 7.6 (6.0, 9.2) |  | 3.3 (1.9, 4.7) |  |
| AD signature cortical thickness |  |  | 0.923 |  | 0.627 |  | 0.795 |
| Below mean | 251 | 3.8 (2.8, 4.8) |  | 7.3 (6.0, 8.6) |  | 3.1 (2.0, 4.3) |  |
| Above mean | 251 | 3.9 (2.9, 4.9) |  | 7.8 (6.5, 9.1) |  | 3.4 (2.2, 4.5) |  |

Note: RBANS = Repeatable Battery for the Assessment of Neuropsychological Status; CI = confidence interval; AD = Alzheimer’s disease; NART = National Adult Reading Test. Estimates were adjusted for age, sex, education level, *APOE*-ε4 carriage and initial RBANS level, where applicable.

**Supplementary Table 2. Associations of MRI parameters, age, initial RBANS score, NART score and test-retest time interval (all modelled as continuous variables) with RBANS practice effects**

| **Characteristics** | **Increase of total scale** | | **Increase of immediate memory index** | | **Increase of delayed memory index** | |
| --- | --- | --- | --- | --- | --- | --- |
|  | **β (95% CI)** | ***P* value** | **β (95% CI)** | ***P* value** | **β (95% CI)** | ***P* value** |
| Hippocampal volume (scaled) | -0.1 (-1.0, 0.8) | 0.842 | 0.2 (-0.9, 1.3) | 0.696 | 0.5 (-0.5, 1.5) | 0.301 |
| Whole brain volume (scaled) | -0.0 (-1.6, 1.6) | 0.987 | -1.3 (-3.3, 0.8) | 0.227 | -0.6 (-2.4, 1.2) | 0.513 |
| Ventricular volume (scaled) | 0.4 (-0.5, 1.4) | 0.353 | 0.5 (-0.6, 1.7) | 0.365 | 0.2 (-0.9, 1.2) | 0.728 |
| AD signature cortical thickness (mm) | 0.7 (-5.6, 6.9) | 0.836 | 5.1 (-2.8, 13.1) | 0.207 | -1.9 (-8.9, 5.1) | 0.589 |
| Age (year) | -0.1 (-0.2, 0.1) | 0.338 | 0.1 (-0.1, 0.3) | 0.179 | 0.1 (0.0, 0.3) | 0.097 |
| Initial RBANS score | -0.1 (-0.2, -0.1) | <0.001 | -0.3 (-0.4, -0.3) | <0.001 | -0.4 (-0.5, -0.3) | <0.001 |
| NART score | -0.1 (-0.2, 0.1) | 0.351 | -0.1 (-0.2, 0.1) | 0.204 | -0.1 (-0.3, 0.0) | 0.060 |
| Test-retest time interval (month) | 0.3 (-0.5, 1.0) | 0.496 | 0.4 (-0.6, 1.4) | 0.469 | -0.1 (-0.9, 0.8) | 0.904 |

Note: MRI = magnetic resonance imaging; RBANS = Repeatable Battery for the Assessment of Neuropsychological Status; NART = National Adult Reading Test; CI = confidence interval; AD = Alzheimer’s disease. Estimates were adjusted for age, sex, education level, *APOE*-ε4 carriage and initial RBANS level, where applicable.

**Supplementary Table 3. Differences between test and retest performance in RBANS after excluding 52 participants with test-retest interval over 6 months**

| **RBANS score,** $\bar{\boldsymbol{x}}$ **± SD** | **Test** | **Retest** | **Difference score** | **Cohen’s d_z_** | ***P* value** |
| --- | --- | --- | --- | --- | --- |
| Total scale | 103.0 ± 11.8 | 107.0 ± 12.8 | 4.0 | 0.53 | <0.001 |
| Immediate memory index | 101.8 ± 12.9 | 109.5 ± 13.2 | 7.6 | 0.73 | <0.001 |
| Delayed memory index | 100.9 ± 10.1 | 104.4 ± 10.5 | 3.5 | 0.38 | <0.001 |
| Visuospatial construction index | 95.7 ± 14.3 | 97.2 ± 14.2 | 1.4 | 0.10 | 0.025 |
| Language index | 104.2 ± 11.2 | 104.7 ± 12.7 | 0.5 | 0.04 | 0.401 |
| Attention index | 109.2 ± 14.3 | 109.6 ± 15.0 | 0.4 | 0.04 | 0.418 |

Note: RBANS = Repeatable Battery for the Assessment of Neuropsychological Status; SD = standard deviation. *P* values were calculated by paired *t* tests.

**Supplementary Table 4. Associations between amyloid status and magnitude of RBANS practice effects after excluding 52 participants with test-retest interval over 6 months**

| **Characteristics** | **No. of participants** | **Increase of total scale (95% CI)** | ***P* value** | **Increase of immediate memory index (95% CI)** | ***P* value** | **Increase of delayed memory index (95% CI)** | ***P* value** |
| --- | --- | --- | --- | --- | --- | --- | --- |
| Amyloid status |  |  | 0.587 |  | 0.040 |  | 0.147 |
| Aβ+ | 222 | 3.8 (2.8, 4.9) |  | 6.6 (5.3, 7.9) |  | 2.8 (1.7, 4.0) |  |
| Aβ- | 228 | 4.3 (3.2, 5.3) |  | 8.6 (7.3, 9.9) |  | 4.1 (2.9, 5.2) |  |

Note: RBANS = Repeatable Battery for the Assessment of Neuropsychological Status; CI = confidence interval. Estimates were adjusted for age, sex, education level, *APOE*-ε4 carriage and initial RBANS level.

**Supplementary Table 5. Associations between amyloid status and magnitude of RBANS practice effects after additionally adjusting for test-retest time interval and modality of amyloid**

| **Characteristics** | **No. of participants** | **Increase of total scale (95% CI)** | ***P* value** | **Increase of immediate memory index (95% CI)** | ***P* value** | **Increase of delayed memory index (95% CI)** | ***P* value** |
| --- | --- | --- | --- | --- | --- | --- | --- |
| Amyloid status |  |  | 0.409 |  | 0.025 |  | 0.068 |
| Aβ+ | 247 | 3.5 (2.5, 4.6) |  | 6.4 (5.1, 7.7) |  | 2.5 (1.3, 3.6) |  |
| Aβ- | 255 | 4.2 (3.2, 5.2) |  | 8.7 (7.3, 10.0) |  | 4.0 (2.9, 5.2) |  |

Note: RBANS = Repeatable Battery for the Assessment of Neuropsychological Status; CI = confidence interval.


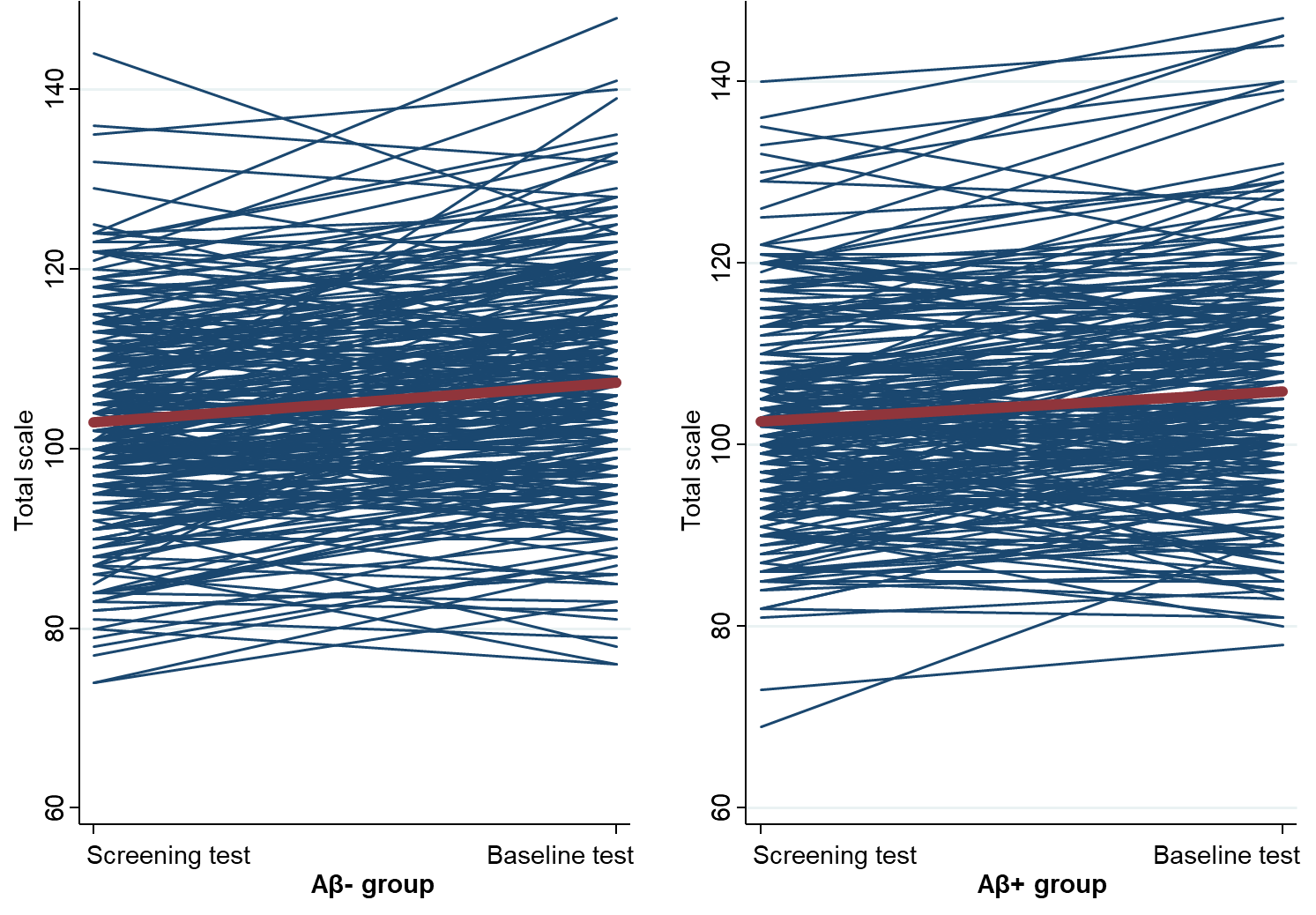


**Supplementary Figure 1. Spaghetti plot of practice effects in RBANS** **total scale by amyloid status**


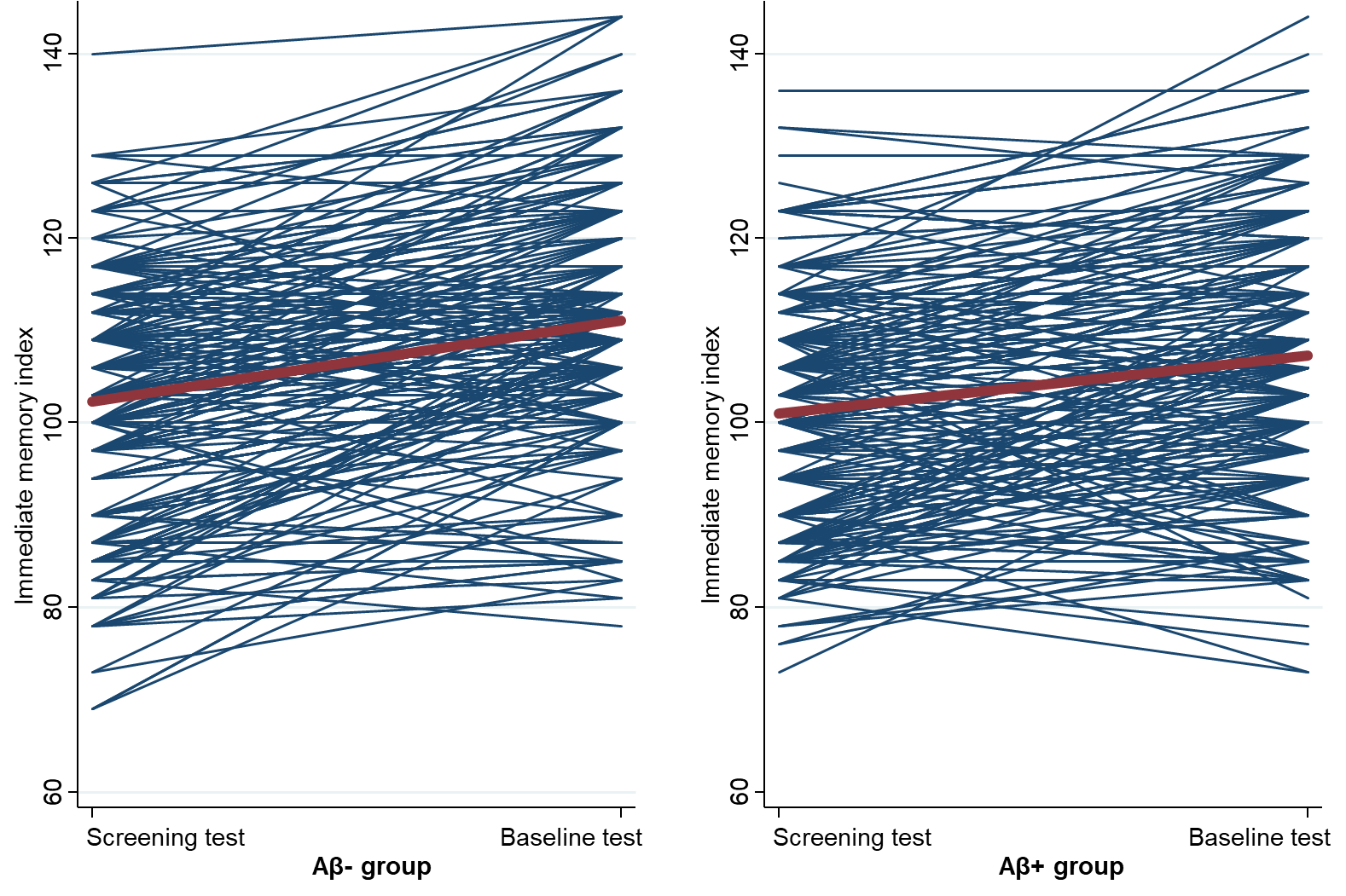


**Supplementary Figure 2. Spaghetti plot of practice effects in RBANS** **immediate memory index by amyloid status**

**
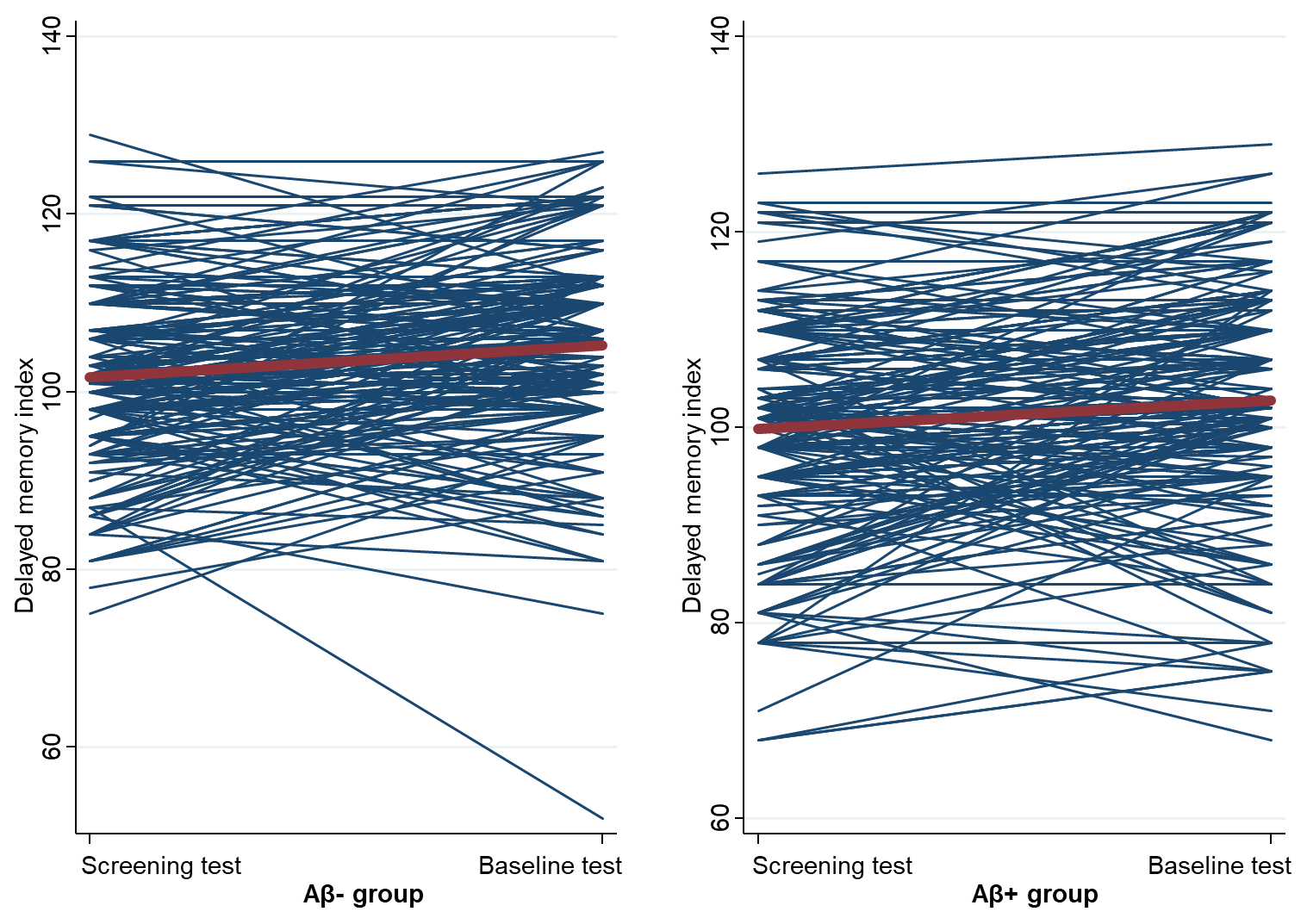
**

**Supplementary Figure 3. Spaghetti plot of practice effects in RBANS delayed memory index by amyloid status**
